# Atypical presentations of primary acquired hypothyroidism – a case series

**DOI:** 10.1186/s12902-023-01488-y

**Published:** 2023-11-06

**Authors:** R. R. Pravin, Sheau Yun Kan, Ser Yee Soh, Daniel Chan, Rashida Farhad Vasanwala

**Affiliations:** 1 General Paediatrics, KK Women’s & Children’s Hospital, Singapore, Singapore; 2 Yong Loo Lin School of Medicine, National University of Singapore, Singapore, Singapore; 3 Endocrinology Service, KK Women’s & Children’s Hospital, Singapore, Singapore; 4 Duke-NUS Medical School, National University of Singapore, Singapore, Singapore; 5 Lee Kong Chian School of Medicine, Nanyang Technological University, Singapore, Singapore

**Keywords:** Hypothyroidism, Thyroid, Growth, Puberty

## Abstract

Primary acquired hypothyroidism in children manifests with a myriad of clinical presentations. Clinical features can be insidious in nature, often under the guise of non-specific presentations to other subspecialties prior to referral to the endocrinologist. Growth failure is a hallmark feature in these children alongside their presenting clinical symptomology which needs to be identified through detailed history, physical examination and analysis of the growth charts. In this case series, we discuss 5 atypical presentations of acquired primary hypothyroidism with multisystemic involvement, including musculoskeletal, hepatobiliary, gynaecological and haematological manifestations. This is of importance as untreated hypothyroidism leads to fatigue, decreased physical activity, suboptimal height gain, disordered puberty and poor neurocognitive development in children with long term detrimental outcomes.

## Introduction

Atypical presentations of acquired primary hypothyroidism are not well described in the paediatric population, although this may be more common than congenital hypothyroidism. Based on limited studies, it has a prevalence of 1:740 (0.135%) as compared to congenital hypothyroidism which has a prevalence of 1:3000–4000 live births (0.027%). [1]. Common aetiologies of acquired primary hypothyroidism include autoimmune thyroiditis, post-thyroidectomy, or iodine deficiency which is more commonly seen in resource-poor countries [2].

Decrease in growth velocity is the most sensitive presentation of this insidious condition, which may also present with a host of other symptoms including development of a goitre, weight gain, myxedema, dry skin, alopecia, cold intolerance, impaired school performance and constipation. Rarer manifestations include pseudoprecocious puberty, coagulopathy presenting as menorrhagia, hyperprolactinemia resulting in pubertal delay and secondary amenorrhoea [3]. It is crucial to identify this condition early, as failure to do so leads to detrimental outcomes such as impaired growth, delayed puberty [4] and poor neurocognitive development [5].

Rare presentations of acquired hypothyroidism have been described in isolated case reports. However, this case series comprises of mostly atypical and typical presentations of acquired primary hypothyroidism. Early identification and initiation of levothyroxine replacement was pivotal in these cases as there was significant clinical improvement noted with catch-up growth and normal neurocognitive development thereafter.

### Case Report 1: Fixing a bone, mending a hormone

**Fig. 1:**
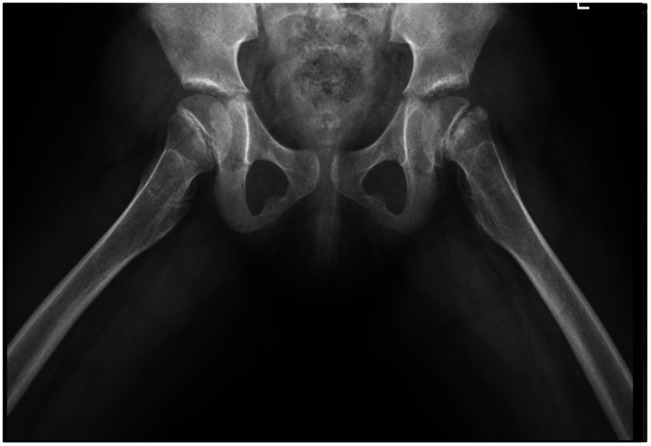
Slipped capital femoral epiphysis of the left hip

The first case describes a 13-year-old girl, who was previously well, presenting with left hip pain for 4 months for which she was admitted under Orthopedics. She developed this pain after playing netball and subsequently developed an antalgic gait. An X-ray radiograph of her hips showed a left slipped capital femoral epiphysis (SCFE) (Fig. 1) for which she underwent screw fixation.

In view of SCFE and high body mass index (BMI), she was referred to the endocrine team for evaluation of endocrinopathy and metabolic health. On further history, she shared she was the shortest girl in class and had not started puberty. She had cold intolerance and chronic constipation. Family history was positive for systemic lupus erythematosus in her mother.

**Fig. 2:**
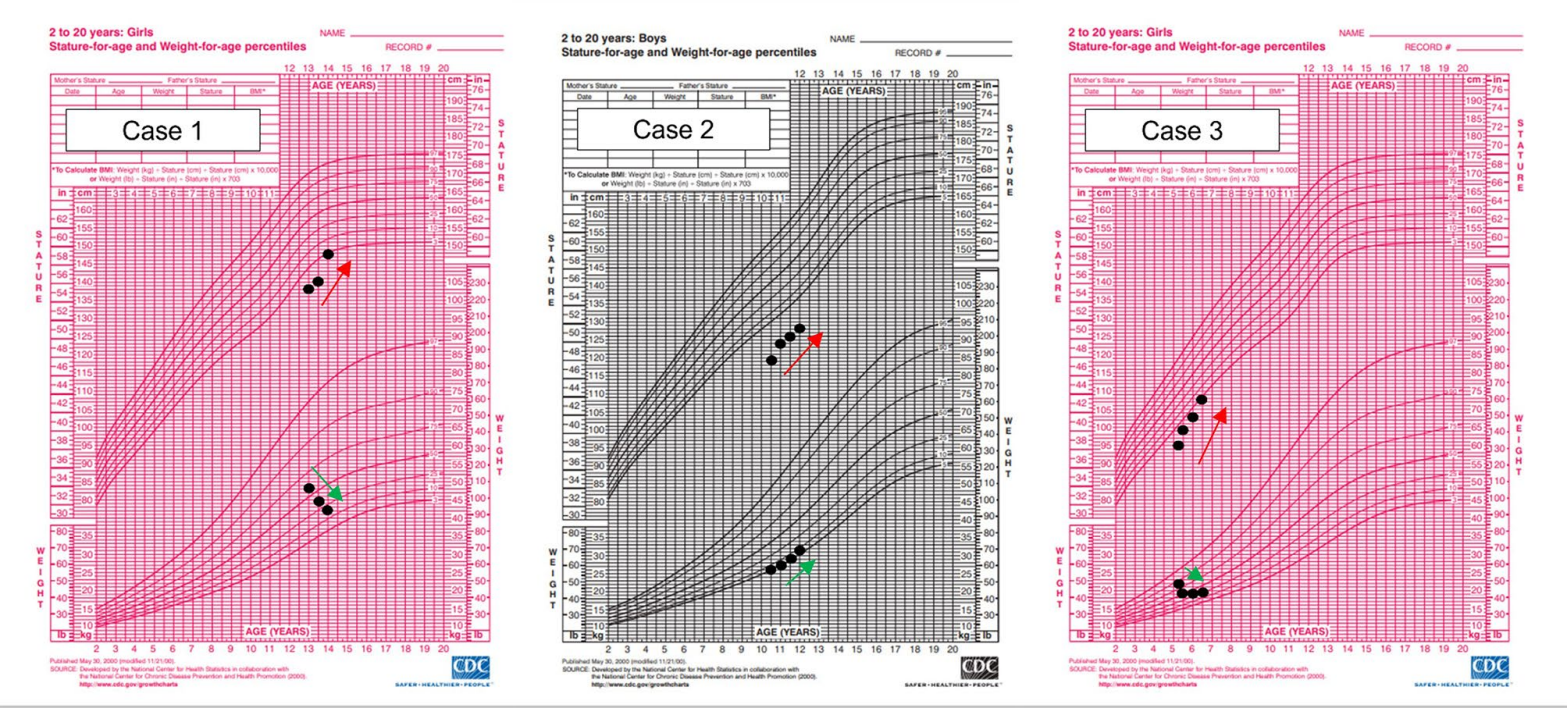
Panel of CDC Growth Charts for cases 1 to 3 (with initial growth points representing centiles at diagnosis and subsequent points representing height acceleration and weight normalization after thyroxine replacement was initiated)

On examination, she was short for age, with a sallow complexion and mild pallor. She had a rounded facies and non-pitting myxedema bilaterally. There was no goitre. Plotting her growth centiles demonstrated that she had growth failure as her height had fallen below the 3rd centile with a steady increase in weight between the 50th to 75th centile. Her growth parameters were as follows: Height 138 cm (SDS − 2.72), Weight 49.1 kg (SDS 0.34) and BMI 25.8 (SDS 1.58). (Fig. 2)

The combination of this clinical presentation with accompanying growth failure prompted further evaluation for hypothyroidism. Thyroid function tests revealed undetectable free thyroxine (FT4), markedly elevated thyroid stimulating hormone (TSH) and thyroid peroxidase antibodies (TPOAb) (Table 1). Bone age was delayed by 4 years 2 months. Putting these clinical, biochemical, and radiographic findings together, the patient was diagnosed with acquired autoimmune hypothyroidism complicated by growth failure and SCFE. She was started on thyroxine replacement with good response in terms of height acceleration, progress of puberty and symptom resolution.

**Table 1:** Summary of cases

Patient No	Gender	Age at presentation	Bone Age	Key presenting feature	First Contact Specialty	Height SDS	Weight SDS	BMI SDS	Thyroid Function		
									FT4 (N:10.3-25.7pmol/L)	TSH (N:0.5-6mU/L)	TPOAb (N:0-60IU/ml)
**Case 1**	F	13 years old	8 years 10 months	SCFE	Orthopaedics	-2.7	0.3	1.6	< 5.4	435.9	144.0
**Case 2**	M	10 years old	6 years 5 months	Myalgia	Rheumatology	-3.3	-1.6	0.6	< 5.2	494.6	124.0
**Case 3**	F	5 years 2 months old	2 years 6 months	Abdominal distension with per-vaginal bleed	Paediatric Surgery	-3.3	1.1	2.8	< 5.2	486.8	388.0
**Case 4**	M	3 years 8 months old	2 years 7 months	Macrocytic Anaemia	Haematology	-3.3	-2.5	0.3	< 5.2	350.8	< 10.0
**Case 5**	F	9 years 3 months old	7 years 9 months	Growth failure	General Practitioner	-1.6	2.3	2.7	< 5.2	458.0	34,910.0

*M: male, F: female, SDS: standard deviation scores, FT4: free T4, TSH: thyroid stimulating hormone, TPOAb: thyroid peroxidase antibodies, SCFE: slipped capital femoral epiphysis

SCFE is a common hip disorder affecting the peripubertal or pubertal adolescent population, with a prevalence of 10.8 cases for every 100,000 children predominantly affecting those between 8 and 15 years of age [6, 7]. Although less common, SCFE can also present in prepubertal children with risk factors of obesity, underlying genetic syndromes such as Down’s syndrome, and untreated hypothyroidism [8, 9]. While they usually present to the orthopaedics department, one must consider endocrinopathies as illustrated in a study by Loder et al. who studied 85 patients with SCFE of whom 40% had hypothyroidism, 25% had growth hormone deficiency and 35% had other disorders such as hypogonadism, hyperparathyroidism, and panhypopituitarism [10]. The thyroid hormone is necessary for maturation of the growth plate chondrocytes [7] without which the stability of the growth plate is compromised, contributing to proximal femoral glide and hence SCFE.

### Case report 2: A mistaken myalgia

A 10-year 6-month-old boy was admitted from the rheumatology clinic for further evaluation of left hip pain with initial impression of reactive arthritis. He complained of generalized myalgia, with arthralgia involving the back, hips, knees, and ankles for the past 3 to 4 months. The pain occurred daily, and was notably worse in the morning. His activities of daily living were affected and he was not able to ambulate comfortably due to the pain. Apart from poorer appetite, there was no prolonged fever or weight loss. He had a recent intercurrent illness with cough and diarrhoea.

On examination, he was short for his age and pale. Cardiopulmonary examination was normal, abdominal examination revealed hepatomegaly. Neurological and musculoskeletal examinations were normal except for a left antalgic gait. There were no rashes. He had no goitre.

**Fig. 3:**
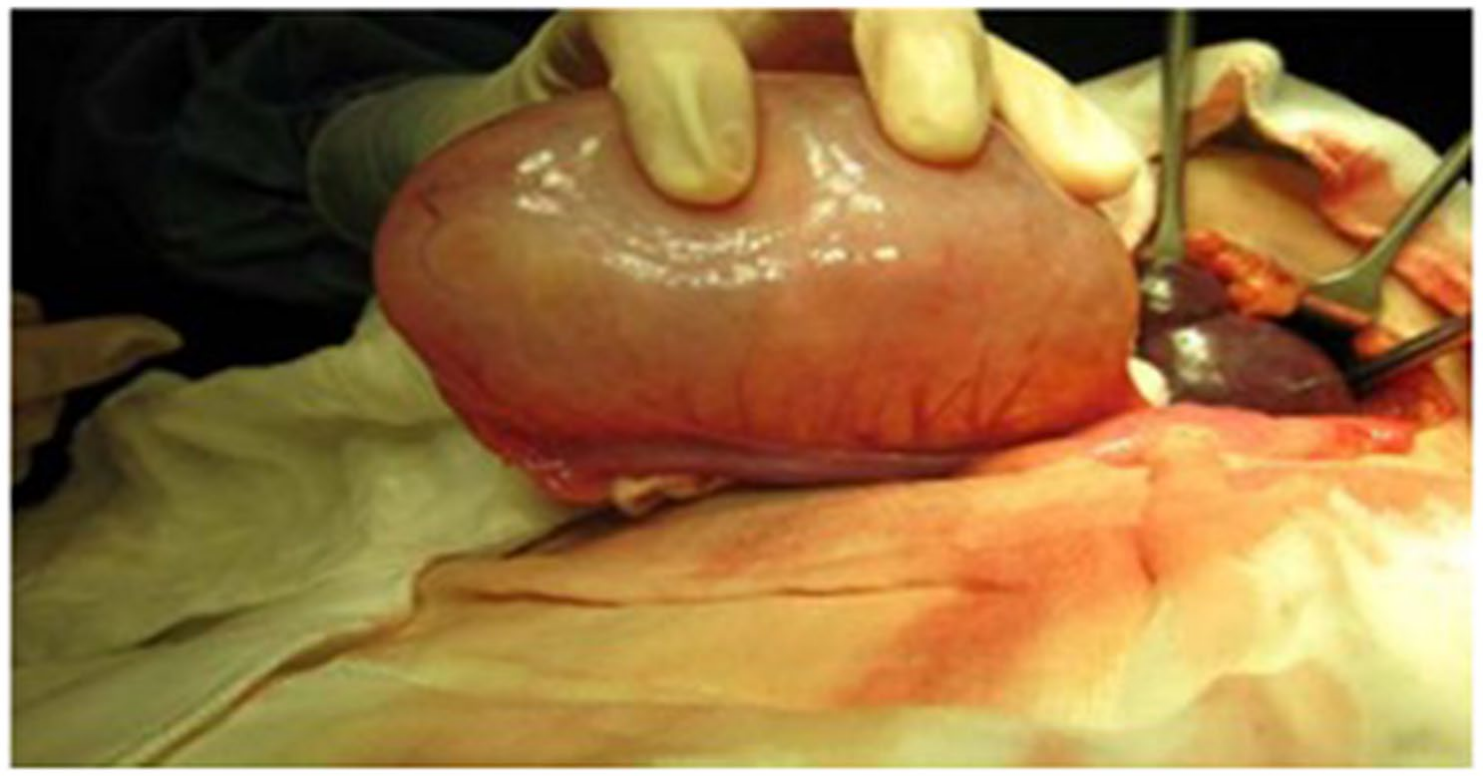
Mega-ovary noted intraoperatively (right)

Plotting his anthropometric measures on an age and gender-appropriate growth chart revealed that he was below the third centile for both height and weight. His height was 119.5 cm (SDS − 3.29), weight of 26.2 kg (SDS − 1.61), and BMI of 18.3 kg/m^2^ (SDS 0.58). (Fig. 3) Further blood investigations showed normochromic normocytic anaemia (Hb 9.3 g/dL), raised erythrocyte sedimentation rate (ESR 56 mm/hr), raised liver and muscle enzymes (alanine aminotransferase 226 U/L, aspartate aminotransferase 270 U/L), creatinine kinase 479 U/L and LDH 965 U/L. Differentials for his non-specific presentation ranged from viral myositis, to autoimmune myopathy with hepatitis, and metabolic myopathy.

In view of his growth failure, bone age was performed which showed severe delay by 4 years 1 month, and this prompted further endocrine workup. Finally, the diagnosis was clinched when his thyroid panel revealed undetectable FT4, elevated TSH and TPOAb levels (Table 1).

The features of generalized muscle ache with elevated liver and muscle enzymes were due to severe untreated primary hypothyroidism. After treatment with thyroxine, his symptoms resolved within 1 month and liver function and muscle enzymes normalized when rechecked in 4 months.

Hypothyroid myopathy rarely presents directly to endocrinologists and may mask itself in various non-specific presenting complaints such as weakness and myalgia. In a study by Duyff et al., 79% of adult patients with newly diagnosed hypothyroidism had muscle weakness although these were not the initial presenting symptoms and can also be seen in hyperthyroidism [11]. The pathogenesis of hypothyroid myopathy is not fully understood, although it is postulated that hypothyroidism leads to abnormal glycogenolysis, mitochondrial oxidative metabolism, and triglyceride turnover which in turn impair muscle function [12]. The clinical manifestations include myalgia, proximal myopathy, stiffness, cramps, elevated muscle enzymes and even rhabdomyolysis [13].

### Case report 3: Not all that bleeds is surgical 

A previously well 5-year 4-month-old girl was admitted under paediatric surgery in view of acute abdominal pain with abdominal distension associated with weight gain and per vaginal bleeding. An immediate tomography scan of the abdomen and pelvis showed bilateral ovarian masses, and she underwent emergency surgery. During the procedure, the surgeon consulted the endocrinologist, due to concerns of enlarged and cystic appearing ovaries in this girl of prepubertal age (Fig. 3). Intra-operatively a right cystic mega-ovary and torsion of the left cystic mega-ovary was noted for which she underwent an emergency left oophorectomy. Post-surgery, an endocrine review was done.

On further history, there was no associated growth concerns. Prior to this episode, she had no evidence of precocious puberty with no breast development or per vaginal bleeding. Parents mentioned she had chronic constipation. Examination revealed she was pre-pubertal with bilateral prominent nipple-areola complexes without breast buds and no signs of virilisation. She had no goitre. Her height was below the third centile and weight was between 90th and 97th centiles. Her height was 95 cm (SDS − 3.33), weight of 22.1 kg (SDS 1.05), and BMI of 24.5 kg/m^2^ (SDS 2.75). (Fig. 3)

In view of cystic ovaries found intraoperatively, a gonadotropin stimulation test was done which showed pre-pubertal levels of follicle stimulating hormone (3.8 IU/L) and peak luteinising hormone (< 0.07 IU/L). Her ovarian tumour markers, (CA-125, alphafetoprotein and beta-HCG) were not elevated. However, her thyroid panel revealed low FT4, elevated TSH and TPOAb (Table 1). Bone age was delayed by 2 years 8 months at a chronological age of 5 years 2 months. Her thyroid ultrasound was normal. The histopathology of the left ovarian specimen showed a multiloculated cyst, with locules ranging from 2 to 5 cm, filled with blood and clots with no identifiable viable tissue. Cytology showed no malignant cells.

Putting together findings of primary hypothyroidism with bilateral cystic ovaries due to ovarian stimulation from elevated thyroid stimulating hormone levels, she was diagnosed to have Van Wyk-Grumbach syndrome. She was treated with thyroxine and on follow-up showed appropriate growth acceleration and resolution of constipation with no recurrence of abdominal symptoms or per vaginal bleeding.

Van Wyk-Grumbach syndrome was eponymously named after Jud Van Wyk and Melvin M. Grumbach and first described in 1960 [14]. The syndrome entails a triad of juvenile hypothyroidism, delayed bone age, and isosexual precocious puberty. High circulating levels of thyroid stimulating hormone directly act on follicle stimulating hormone receptors, and stimulates adenylyl cyclase activity, which in turn stimulates precocious puberty [15]. Thyroid stimulating hormone alpha subunits are similar to those of follicle stimulating hormone and hence excess thyroid stimulating hormones act on the follicle stimulating hormone receptors, triggering a state of precocious puberty [16]. Treatment with thyroxine remains the mainstay for reverting the patient to a prepubertal state, although the patient may still be at risk of developing secondary central precocious puberty due to priming of the hypothalamic pituitary gonadal axis.

### Case report 4: An anaemia like none other

A 3-year 7-month-old boy was referred from the haematologist to the endocrine clinic for concerns of hypothyroidism. This child has a background of STAT-1 gene mutation and was on follow up with the immunologist for a history of severe and frequent infections since young. He was initially referred to the haematologist for persistent macrocytic anaemia detected on full blood count with normal vitamin B12 and folate levels. His haemoglobin was 10.4 g/dL with both an elevated mean corpuscular volume of 94.5 fL and mean corpuscular hemoglobin of 30.7 pg.

He was noted to also have marked speech delay. Further history revealed that he was recently always tired and not attentive in school. His parents remarked he had recent onset constipation. Family history was significant for maternal autoimmune hypothyroidism on thyroxine replacement. On examination, he was noted to be small for age, pale with periorbital and pre-tibial oedema. He had dry skin and cool peripheries, and was noted to have generalised hypotonia. He had no goitre.

**Fig. 4:**
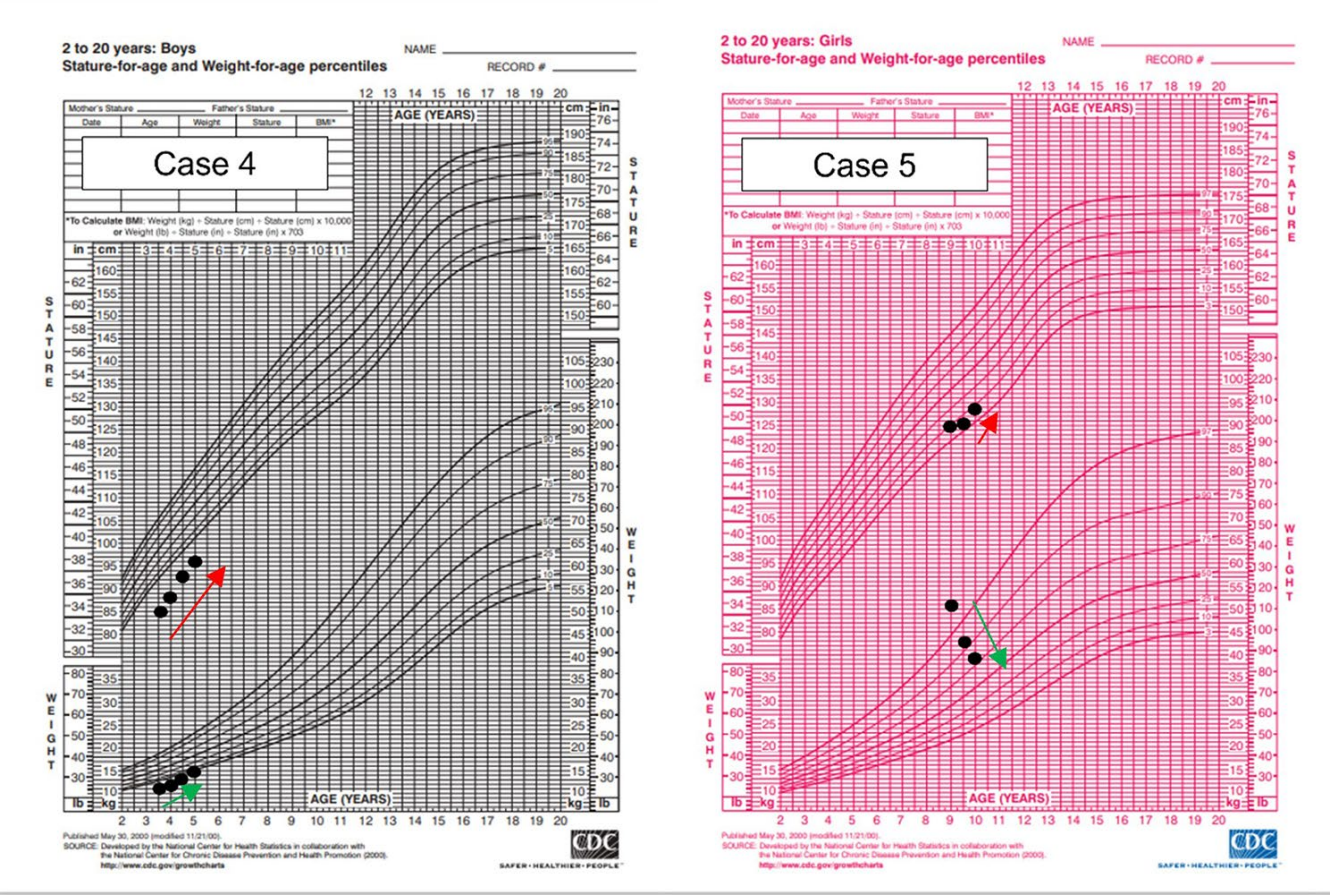
Panel of CDC growth charts for cases 4 to 5 (with initial points representing centiles at diagnosis and subsequent points representing progress after thyroxine replacement was initiated)

Plotting his centiles on growth charts revealed he was below the third centile for both height and weight. His height was 85.2 cm (SDS − 3.31), weight of 11.5 kg (SDS − 2.52), and BMI of 15.8 kg/m^2^ (SDS 0.3). (Fig. 4) A thyroid panel revealed low FT4, elevated TSH with normal TPOAb levels. Bone age was delayed by one year. A thyroid ultrasound revealed a hypoplastic thyroid gland with diffusely increased echogenicity.

These findings of macrocytic anaemia together of growth failure with developmental delay were due to severe untreated primary hypothyroidism. Eventually, macrocytosis on his full blood count resolved without any haematinics with improvement in growth and development once thyroxine was promptly started.

Macrocytic anaemia that is not responsive to haematinics, with normal vitamin B12 and folate levels, should prompt the physician to assess for other less common causes, such as hypothyroidism. Hypothyroidism can present with macrocytic anaemia possibly due to physiologic adaptation of decreased tissue oxygen requirements secondary to a decrease in basal metabolic rate. In these patients, plasma erythropoietin levels have been shown to be low. Furthermore, the degree of anaemia may correlate with the severity of hypothyroidism [17].

Of note, this child also had STAT-1 gene mutation and there has been a mechanistic relationship described between this mutation and the development of hypothyroidism. STAT-1 gene is a signal transducer and activator of transcription 1 and this is important for regulation of immune system function [18]. However this gene also has an effect beyond the immune system, as thyroid morphology and function can be profoundly affected by the absence of STAT-1 due to significant disruption of follicular architecture and thyrocytes, resulting in decreased serum thyroxine levels [19]. Hence, in children with underlying immunologic or genetic disorders presenting with endocrinopathies, it is pivotal to determine if there is any association between the two.

### Case report 5: A short story

A 9-year-old girl was referred from the general practitioner to the endocrinology clinic for growth concerns. She was adopted since 3 days of age, hence there was no information regarding her biological parental height. She was one of the tallest in her class when she was 2 year 6 months old in preschool. However, by the end of 7 years old, her parents noted she was gaining weight but not growing taller. Plotting her growth measurements on age and gender-appropriate growth charts revealed she was between 3rd to 10th centile for height and more than 97th centile for weight. Height was 124.2 cm (SDS −1.63), weight 51.5 kg (SDS + 2.27), and BMI was 33.4 kg/m^2^ (SDS + 2.68). (Fig. 4) On examination, she was obese with abdominal obesity but had no features of Turner syndrome or other dysmorphic features. Cardiovascular, respiratory, abdominal examinations were unremarkable. There was no goitre found on neck examination. There were no Cushingoid features.

A screen for thyroid function revealed low FT4, elevated TSH and TPOAb (Table 1). Bone age was delayed by one and a half years as compared to her chronological age. Thyroid ultrasound showed a structurally normal gland with no heterogeneous echotexture.

Her biochemical and auxologic features were in keeping with autoimmune primary hypothyroidism causing significant growth failure and predominantly endogenous obesity contributed by hypothyroidism. Her metabolic screen comprising glycated hemoglobin, lipid panel, and liver function tests were unremarkable. She was started on thyroxine with significant catch-up growth.

Declining height velocity resulting in short stature is the most common manifestation of hypothyroidism. Growth delay may be the only presenting complaint before other symptoms occur, highlighting the importance of having a high index of suspicion for hypothyroidism for any child that presents with declining height velocity, who may first present incidentally to the primary care physician. Hypothyroidism leads to delayed skeletal maturation and therefore a delayed bone age. Prolonged, untreated hypothyroidism would result in permanent height deficits [4]. It is important for general practitioners to identify this early especially during general health screenings of well children.

## Discussion

While autoimmune thyroiditis remains the commonest cause of primary acquired hypothyroidism [20], a myriad of rare but interesting presentations in the paediatric population have been reported across various case reports over the years. These unique presentations are related either to untreated hypothyroidism directly or resultant downstream complications. These can range from mild manifestations such as hypertrichosis [20] or musculoskeletal complications such as SCFE. An isolated case report suggested a possible association between hypothyroidism and limb anomalies such as oligosyndactly in a child diagnosed with Van-Wyk Grumbach syndrome [21], alluding to a similar phenomenon of thyroid disorders reported in other syndromes with musculoskeletal anomalies such as Cenani-Lenz syndactly syndrome which was shown to be associated with congenital hypothyroidism [22].

More significant manifestations such as rhabdomyolysis, acute compartment syndrome or muscular pseudohypertrophy seen in syndromes such as Koche-Debre-Semelaigne syndrome or Hoffman’s syndrome caused by hypothyroid myopathy have also been reported in both children and adults [12]. Other systems involved can range from haematological derangements, such as macrocytic anaemia, to gastrointestinal symptoms such as chronic abdominal pain reported in children with hypothyroidism, not necessarily related to constipation [23]. Life-threatening complications such as pericardial effusion can also result from hypothyroidism which should be in the list of differentials considered and emergently dealt with [24].

The symbiotic relationship between the central nervous system and endocrine systems is evident in the pituitary-hormone axis. Hence, derangements in thyroid hormone levels can affect other aspects of this axis. Precocious puberty secondary to hypothyroidism can present as vaginal bleeding in girls of prepubertal age, and likewise macro-orchidism in young boys in the absence of other clinical signs of virilisation [25]. Pituitary hyperplasia has also been reported in cases of hypothyroidism alongside other manifestations described in this article [20]. Isolated studies have also suggested a possible association between hypothyroidism and pathologic myopia in children, with improvement in progress of myopia with initiation of treatment [26, 27]. Literature has shown that children can present with more than one of these rare clinical signs and symptoms, and a simple thyroid function test can help clinch the diagnosis in these diagnostic dilemmas.

Although this case series does not offer an exhaustive list of all atypical presentations, it aims to more importantly offer an insight to the presentations physicians may encounter in their daily practice. Acquired primary hypothyroidism can present to the general paediatrician or a subspecialist, guised under a spectrum of presentations. Clinical acumen is required to decipher the child with an underlying thyroid endocrinopathy as leaving it undiagnosed has its consequences such as linear growth failure and impact on neurodevelopmental and physical capabilities [28]. It is important to also highlight that while the presence of a goitre alerts clinicians to the possibility of a thyroid issue, the absence of one does not preclude the diagnosis of hypothyroidism and could have contributed to the delay between onset and diagnosis across these cases. Appropriate history taking and physical examination, in correlation with growth parameters, need to be performed with relevant investigations to diagnose patients in a timely manner, with early initiation of treatment reversing the underlying pathology and its associated complications.

All 5 patients and families reported improved outcomes in terms of overall improvement in physical well-being, growth and neurocognitive outcomes upon serial follow-up from diagnosis to the point of publication. Table 2 shows details of all 5 cases based on their latest follow-up including their growth centiles and pubertal stages.

**Table 2:** Follow-up of cases

Patient No	Gender	Age at latest follow up	Diagnosis	Height SDS	Weight SDS	BMI SDS	Pubertal Status				Latest Thyroid Function Test Results		Latest Dose of Thyroxine
							B	PH	G	Age of onset of menarche (years)	FT4 (N:10.3-25.7pmol/L)	TSH (N:0.5-6mU/L)	
**Case 1**	F	15 years 7 months old	SCFE	-1.2	0.7	1.2	3/3	2	NA	14	13.1	1.1	100mcg OM
**Case 2** ^**†**^	M	21 years 6 months old	Hypothyroid Myopathy	-2.4	-2.1	-0.9	NA	4–5	4–5 Testes 15ml/15ml	NA	< 5.4	193.1	175mcg OM
**Case 3**	F	17 years 8 months old	Van Wyn Grumbach Syndrome	-2.0	0.7	1.4	ND	ND	NA	11	12.8	7.1	100mcg OM
**Case 4**	M	10 years 8 months old	STAT-1 immunodeficiency syndrome	-1.3	-2	-1.8	NA	1	1	NA	17.5	1.8	50mcg OM (Weekdays) 75mcg OM (Weekend)
**Case 5**	F	13 years 2 months old	Autoimmune Hypothyroidism	-0.8	1.7	2.0	3+/3+	1+	NA	11	10.3	14.6	125mcg OM

*M: male, F: female, SDS: standard deviation scores, FT4: free T4, TSH: thyroid stimulating hormone, Tanner Staging: B: Breast, PH: Pubic Hair, G: Genitalia, SCFE: slipped capital femoral epiphysis, OM: once every morning, NA: not applicable, ND: not documented. ^**†**^Deranged thyroid function due to poor compliance to medications

## Conclusion

Acquired primary hypothyroidism manifests as a spectrum of both acute and insidious presentations. A comprehensive history, careful physical examination, detailed plotting and longitudinal review of a child’s growth trajectory are key to raising clinical suspicion, even before a thyroid function test confirms biochemical hypothyroidism. Subsequent initiation of thyroid hormone replacement leads to improvement and clinical resolution of symptoms with good catch-up height gain and optimised neurocognitive development.

## Data Availability

All data are available at request from the corresponding author.
